# Probiotic Yoghurts with Sea Buckthorn, Elderberry, and Sloe Fruit Purees

**DOI:** 10.3390/molecules26082345

**Published:** 2021-04-17

**Authors:** Dorota Najgebauer-Lejko, Katarzyna Liszka, Małgorzata Tabaszewska, Jacek Domagała

**Affiliations:** 1 Department of Animal Product Technology, University of Agriculture, 30-149 Krakow, Poland; liszka.katarzyna@op.pl (K.L.); jacek.domagala@urk.edu.pl (J.D.); 2 Department of Plant Product Technology and Nutrition Hygiene, University of Agriculture, 30-149 Krakow, Poland; malgorzata.tabaszewska@urk.edu.pl

**Keywords:** probiotic yoghurt, elderberry, sea buckthorn, sloe, antioxidant properties, starter bacteria, texture, color, aromatic compounds, sensory analysis

## Abstract

Elderberries, sea buckthorn, and sloe berries are fruits of wild-grown bushes, valued in folk medicine for their health-promoting properties but still rarely applied in food. The aim of the present study was to produce probiotic yoghurts with a 10% addition of sweetened purees prepared from elderberries (EPY), sea buckthorn (SBPY), and sloe berries (SPY) and to assess their chemical composition, acidity, content of polyphenols and anthocyanins, ferric reducing antioxidant power (FRAP) and antiradical power (ARP), level of starter microbiota, concentration of acetaldehyde and diacetyl, syneresis, instrumentally measured color and texture parameters, and sensory acceptance. The results were compared to those obtained for plain probiotic yoghurt (PPY) and the changes tracked during 1 month of cold storage at 2 week intervals. The addition of elderberry and sloe berries significantly increased the antioxidant capacity of probiotic yoghurts, probably due to a high content of polyphenols, especially anthocyanins. However, anthocyanins were more stable in the EPY when compared to the SPY. All yoghurt treatments were characterized by good sensory quality and viability of starter microorganisms, including probiotic strains during cold storage. Elderberries promoted the evolution of diacetyl in yoghurts during storage and, together with sloe berries, produced increased syneresis and the greatest changes in color profile compared to PPY.

## 1. Introduction

Yoghurt is the most popular fermented milk product, consumed not only for its taste but also for its nutritional and health-promoting properties. It is produced from a milk base with standardized fat and total solid content which is homogenized, pasteurized, cooled, and inoculated with two starter bacteria, i.e., *Streptococcus thermophilus* and *Lactobacillus delbrueckii* ssp. *bulgaricus*, before being left to coagulate in a vat or directly in cups. Usually about 4.5 cups with yoghurt are transferred to refrigerated storage after reaching the desired pH, or the yoghurt gel from a vat is stirred, cooled to about 20 °C, and mixed with flavoring (e.g., fruit) preparation before cold storage [1]. The processing methods for yoghurt manufacture may be modified, whereas different ingredients may also be incorporated into the yoghurt formula, such as concentrated dairy ingredients, sugar or other sweeteners, probiotic cultures, stabilizers, flavorings, and many other nutritional and functional components [1,2]. Different types of yoghurts are produced to meet different consumers’ demands. Fruit-flavored yoghurts are more popular than natural (plain) ones. The most popular fruit flavors are strawberry, peach, raspberry, blueberry, lemon–lime, cherry, and mixed berry [3]. Producers outdo each other in developing new flavors and new products to broaden their product portfolios in order to attract consumers and to satisfy even the most challenging consumer demands. Currently, not only are consumers looking for new tastes and flavors, but they are also paying attention to the origin and bioactive properties of foodstuffs as they become more and more conscious of how their diet affects their health and wellbeing. Nevertheless, yoghurt production with the addition of wild-grown fruit is still an under–explored area. To the best of our knowledge, there are only a few reports on yoghurts supplemented with sea buckthorn berries [4,5,6,7] and none devoted to the application of sloe berries. Elderberry, however, is added to yoghurt, but only in small concentrations, in order to enhance the dark reddish-violet color of, e.g., blueberry or forest fruit yoghurt, not as flavoring or a bioactive ingredient itself. Elderberry, the fruit of black elder (*Sambucus nigra* L.) shrub, and its preparations (powders, extracts, concentrates) are used as a potent source of natural colorants because they contain considerable amounts of anthocyanins, mainly cyanidin glycosides [8,9]. Moreover, anthocyanins and other phenolic compounds, such as flavonols and phenolic acids from elderberries, are known for their excellent free-radical-scavenging ability [10]. Due to a high content of dietary phytochemicals, as well as antioxidant, antibacterial, antiviral, immunostimulating, and antiallergic properties, both elderberries and elderflowers have been used for centuries in food production and in folk medicine to treat different ailments [10].

Sloe (blackthorn) berry is the fruit of the blackthorn bush (*Prunus spinosa* L.), a plant from the Rosaceae family which grows wildly in different parts of Europe. Sloe berries have been used in folk medicine for different purposes, such as to cure flu, cold, diabetes, or cardiovascular diseases [11,12]. The fruits are rich in many bioactive components such as flavonoids, anthocyanins, phenolic acids, vitamins, minerals, organic acids, and many other ingredients with antioxidant and antimicrobial activity [11,12]. Ürkek et al. [12] revealed that sloe berries can be added to foodstuffs, e.g., ice cream as colorants, flavorings, and antioxidant additives, as well as a viscosity increasing agent.

**Figure 1:**
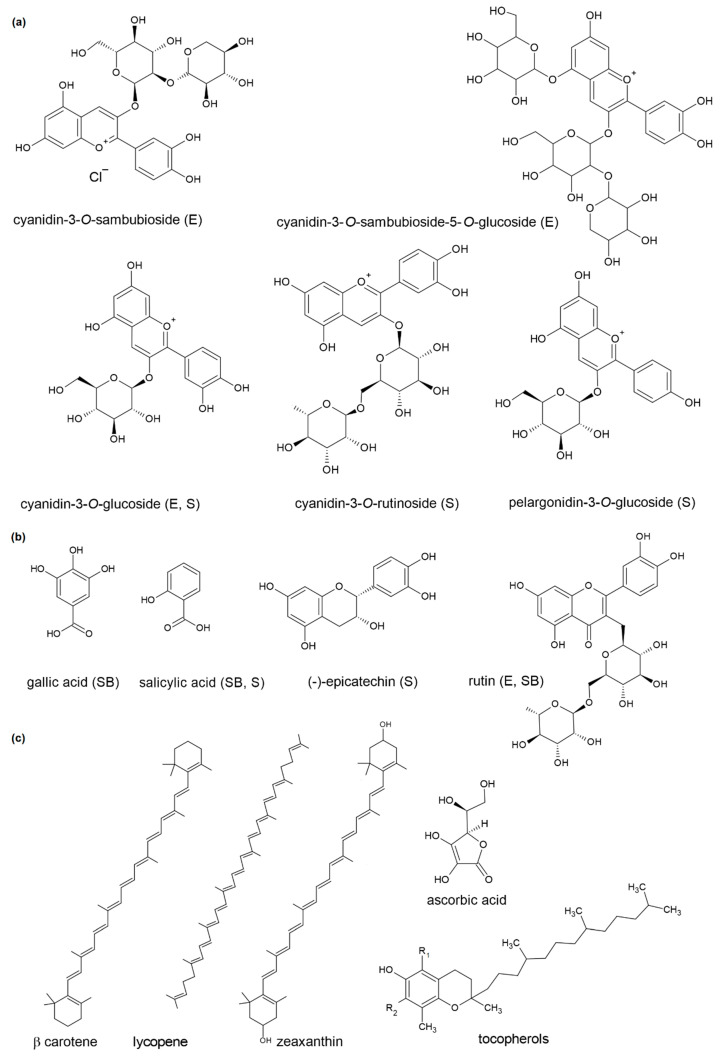
Structures of the most important antioxidants present in sea buckthorn berries (SB), elderberries (E), and sloe berries (S): (**a**) anthocyanins; (**b**) phenolic acids and flavonoids, (**c**) other antioxidant substances from sea buckthorn berries.

In contrast to the dark-colored elderberries and sloe berries, the fruits of sea buckthorn (*Hippophae rhamnoides* L.) are yellow–orange due to a high concentration of carotenoids. They also contain considerable amounts of other natural antioxidants and bioactive compounds, such as ascorbic acid, tocopherols, vitamins B, K, and A, flavonoids, fatty acids, β-sitosterol, minerals, organic acids, amino acids, and carbohydrates. A relatively high total lipid content and the rich, unique profile of aromatic compounds are distinctive for sea buckthorn fruits [5,13]. The structures of major antioxidant substances present in elderberries, sea buckthorn, and sloe berries are shown in Figure 1.

All the mentioned fruits have great potential as supplements for foodstuffs such as yoghurt, in which they may serve as antioxidants and natural alternatives for synthetic coloring and flavoring agents. Thus, the aim of the present work was to develop and produce probiotic yoghurts with the addition of fruit purees prepared from sea buckthorn berries, elderberries, and sloe berries and to analyze their selected properties, including basic composition, content of selected antioxidant substances and antioxidant capacity, level of starter microbiota (including probiotic strains) and yoghurt-specific aromatic compounds, color profile, and texture during cold storage. We believe that the results of the study would be helpful in developing novel dairy products with functional properties with the use of rarely applied fruit components with great health-promoting potential.

## 2. Results

### 2.1. Chemical Composition of Fruit Purees and Probiotic Yoghurts

Out of all the studied fruit preparations, elderberry puree was the richest in phenolic compounds, followed by sloe and sea buckthorn fruit (Table 1). The content of anthocyanins was similar in both dark fruit preparations and was almost fourfold higher than in the sea buckthorn puree. Moreover, anthocyanins constituted about 19% of all phenolic compounds in elderberry puree and almost 29% of phenolics in sloe berry puree. HPLC analysis revealed that sea buckthorn fruits contained considerable amounts of phenolic and carboxylic acids, such as salicylic, chlorogenic, caffeic, ferulic, *p*-coumaric, and *t*-cinnamic acids and a high content of (−)-epicatechin, which was not detected in other fruit preparations. All of the mentioned phenolic acids, except for salicylic and *t*-cinnamic acids, were also detected in sloe berries, although in much lower concentrations. Elderberry, however, contained only small amounts of caffeic and *p*-coumaric acids. The analyzed fruit purees also contained flavonoids; myricetin was detected in sea buckthorn and sloe, whereas rutin and quercetin were present in elderberry puree. The latter fruit preparation also contained a significant amount of (+)-catechin.

The basic chemical compositions of all the analyzed probiotic yoghurt treatments are shown in Table 2. All fruit yoghurts were characterized by a higher total solid, carbohydrate, and fiber content when compared to the plain treatment. In contrast, ash content was the highest in the natural (plain) probiotic yoghurt (PPY). The lowest fat concentration was measured in the sloe berry yoghurt (SPY). On the other hand, however, this product was the most abundant in the dietary fiber fraction, which was almost threefold higher than in other fruit treatments and more than sixfold higher than in the PPY. Protein concentration was unaffected by the yoghurt type and exceeded 4%, which is essential in order to obtain the proper structure of yoghurt gel.

### 2.2. Antioxidant Substances and Capacity

The obtained results indicate that the total phenolic content (TPC) in yoghurts determined using the Folin–Ciocalteu method was strongly dependent on the type of fruit puree added; the highest values were stated for elderberry probiotic yoghurt (EPY), followed by the SPY (Table 3). Similarly to the puree, sea buckthorn berry fruit yoghurt (SBPY) was characterized by the lowest concentration of phenolic compounds, whereas plain yoghurts, as expected, contained hardly any phenols. SBPY contained, on average, a 23–40% lower amount of phenolic substances than EPY and 69–82% of the total phenolic content found in SPY. The amount of phenolics decreased during storage, especially for products with dark fruits (elderberries and sloe berries). Similar relationships were found in the case of total monomeric anthocyanin content (TMAC). However, the concentrations of these compounds were more stable during storage, except for SPY, which contained approximately 50% of the initial anthocyanins level at the end of the storage period. Out of all the treatments, EPY was the most abundant in TMAC. Very small amounts, practically negligible, of TMAC were detected in SBPY. Moreover, SBPYs were characterized by a similar ferric reducing antioxidant power (FRAP) to the plain treatment. In contrast, at least fourfold higher values of FRAP were determined during analyses of EPY and SPY. Antioxidant capacity measured as ferric reducing ability was stable during the whole cold storage period of all the analyzed products. The ability to scavenge DPPH radicals (ARP—antiradical power value) of the SBPY was twofold but insignificantly (*p* > 0.05) higher than that of natural yoghurt, whereas the addition of dark fruits resulted in significantly (*p* ≤ 0.05) higher ARP values. Furthermore, in this case, no significant changes in antioxidant capacity were observed during 1 month of storage, except for SPY where a significant drop in the ARP value was observed but only after 2 weeks of the experiment.

### 2.3. Acidity and Microbiological Quality

The results of titratable acidity, pH, and count of starter bacteria determined in the analyzed yoghurt treatments are shown in Table 3. Titratable acidity of yoghurts was in the range of 0.81–1.15% lactic acid. Sea buckthorn and blackthorn fruit additives resulted in higher acidity of fresh probiotic yoghurts when compared to other treatments but these differences were insignificant after storage. The former treatments also produced yoghurts with the lowest pH directly after production. Active and titratable acidity of the analyzed fruit products changed only slightly and insignificantly (*p* > 0.05) during storage in comparison to the non-supplemented treatment, for which the post-acidification effect could be observed with a rise in acidity equal to 0.25% lactic acid and drop in pH by 0.25 units.

No statistically important effect of yoghurt type and storage time was observed regarding all starter microorganisms. The number of bacteria was in the stable range of 10^7^–10^8^ cfu/g for *Lactobacillus acidophilus* La-5, and in the order of magnitude of 10^6^ cfu/g for *Bifidobacterium animalis* ssp. *lactis* BB12 and 10^8^ cfu/g for *Streptococcus thermophilus*. No yeast and molds were detected in all treatments during 1 month of refrigerated storage.

### 2.4. Aromatic Compounds

Diacetyl content in the produced probiotic milks tended to increase during storage, and the most pronounced increase was observed in EPY after 2 and 4 weeks of storage (Table 3). For this treatment, diacetyl concentration after storage was twofold higher than in the non-supplemented PPY. Furthermore, SPY was also characterized by a significantly higher concentration of this aromatic compound after 2 weeks of storage compared to the PPY. Acetaldehyde was another characteristic substance detected in the analyzed dairy products, ranging from 2.38 mg/100 mL in SPY on the first day after production to a value as high as 6.73 mg/100 mLin SBPY on the 15th day of storage (Table 3). There were no significant differences between the natural and fruit yoghurts regarding concentration of acetaldehyde.

### 2.5. Texture and Color Parameters

The average values obtained during the textural study (back extrusion test) and color measurements of the analyzed yoghurts are shown in Table 4. Textural characteristics, such as firmness, consistency, and index of viscosity differed insignificantly between different treatments (*p* > 0.05). All back extrusion attributes measured for all tested fermented milks did not change significantly during storage, although firmness and consistency parameters tended to increase, whereas index of viscosity tended to decrease during storage. Cohesiveness of SBPY and EPY at the end of the storage period reached lower values when compared to PPY (*p* ≤ 0.05).

In contrast to texture, all color parameters were highly dependent on the type of probiotic yoghurt. Average values of lightness (L*) increased in the following order: EPY < SPY < SBPY < PPY. The negative a* value obtained for the PPY indicates that the green color coordinate prevailed over the red one. Addition of all types of fruit preparations shifted the a* variable into the positive range connected with redness. Yoghurt treatments with dark fruits, such as elderberry and sloe berry, were characterized by similar a* values (*p* > 0.05), which was about threefold higher than the respective coordinate determined for SBPY. Yoghurts with dark fruit purees were also characterized by comparable b* values, being in the negative range specific for the blue color component, with a hue angle (*h°*) of approximately 351–354°, which is in the range between blue (270°) and bluish-red (0/360°), and chroma in the range of ~14–17 (C*). On the other hand, SBPYs were more yellowish than all the other treatments. SBPY was also characterized by the lowest hue angle and the highest chroma (which is a measure of color saturation intensity). All color parameters were stable during storage as no significant changes were detected during 4 weeks of the experiment duration.

### 2.6. Sensory Analysis

**Figure 2:**
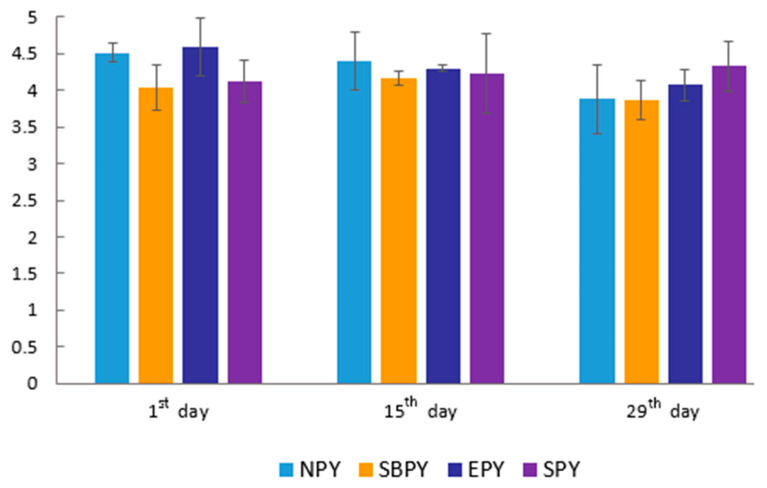
Overall acceptability in sensory evaluation of natural yoghurt (NPY) and yoghurts with sea buckthorn berry puree (SBPY), with elderberry puree (EPY), and with sloe berry puree (SPY) during cold storage.

There were no significant differences between yoghurt treatments in terms of sensory quality (Figure 2). The calculated general scores for overall preference were in the range of 3.87 (SBPY on the 29th day) to 4.51 (NPY on the first day) on a five-point scale. The components of sensory quality, such as color, taste, consistency, and general appearance, received similar scores for all the tested products (data not shown). Only the odor was less desirable in SBPY when compared to other treatments, and only a slight and insignificant decrease in sensory quality was observed during 29 days of cold storage. This indicates that all yoghurts were characterized by a good sensory quality.

## 3. Discussion

### 3.1. Acidity and Aromatic Compounds

Lactic acid is the main flavor component produced by starter bacteria during yoghurt production and storage. This compound is responsible for specific acidity of fermented milks, i.e., the main taste sensation perceived during their consumption [14]. Probiotic yoghurt produced and analyzed by Baranowska et al. [15], containing the same starter bacteria composition as in the present study, contained 0.88% (fresh) to 1.01% (after 14 days of storage) lactic acid. This range is almost the same as the results obtained for natural ABT yoghurt in the present study. Higher initial titratable acidity (and lower pH) values obtained for fruit yoghurts may result from the acidity of fruits itself. Values of pH measured for fruit purees, equal to 2.93 ± 0.09, 3.54 ± 0.02, and 3.20 ± 0.04 (data not shown), respectively, for sea buckthorn, elderberry, and sloe berry, confirm the above statement.

A post-acidification effect, i.e., a significant increase in titratable acidity, could be observed but only in the case of natural yoghurt. Titratable acidity and pH in fruit yoghurts remained almost unchanged during 1 month of cold storage. This means that the lactic-acid-producing activity of starter bacteria was somehow stopped but not the whole metabolic activity as, e.g., in the elderberry treatment, where elevation of the diacetyl production was observed during storage. Diacetyl in fermented milks is produced mainly through the action of lactic acid bacteria (LAB) which metabolize citrates [14,15]. Elderberries are reported to contain considerable amount of citric acid, which could serve to probiotic yoghurt bacteria as a substrate for the production of the abovementioned aromatic compound. According to Veberic et al. [16], elderberry fruit is exceptionally rich in citric acid, containing from 3.11 to 4.81 g/kg FW of this organic acid, when compared to other fruits, e.g., apple, sweet cherry, and sour cherry (0.07–0.54 g/kg FW citric acid).

According to Cirlini et al. [17], lactic acid bacteria are able to ferment elderberry juice with lactic acid being the main product of bacteria metabolism. However, this study revealed that, in contrast to fermented milk, the *Lactobacillus* strains used in the elderberry juice environment utilized mainly organic acids (malic and citric acids) instead of sugars.

In addition to lactic acid, acetaldehyde, diacetyl, acetone, and acetoin are the main aromatic compounds found in yoghurt. There are several factors that affect their levels in the products, as well as those of other aroma-contributing substances, including composition and activity of starter cultures, milk source, processing techniques and parameters, and additives, e.g., stabilizers and flavors [14]. Tamime and Robinson [2] reported the following average levels of acetaldehyde and diacetyl concentrations produced by *Streptococcus thermophilus* and *Lactobacillus delbrueckii* ssp. *bulgaricus* in traditional yoghurt: 2.0–41.0 and 0.4–0.9 μg/g, respectively. In our study, acetaldehyde content was in the lower scope of the abovementioned range, whereas the concentration of diacetyl was much higher. However, our yoghurt was produced using different LAB cultures, which is one of the key factors influencing the aroma profile. Therefore, the results of Baranowska et al. [15] are more relevant for comparison as they reported a 7.12–10.08 mg/L content of diacetyl (with an increase in concentration measured during 2 weeks of storage), as well as acetaldehyde concentration fluctuation from 16.06 mg/L in fresh to below 10 mg/L in stored natural ABT yoghurt. Taking these ranges into consideration, we obtained lower levels of both diacetyl and acetaldehyde. However, in this case, discrepancies could have resulted from other factors such as differences in processing, raw materials, and the proportions of *Lb. acidophilus*, *Bifidobacterium* ssp., and *Str. thermophilus* used to ferment milk.

With regard to the effect of fruit addition, the present results are in agreement with the study of Ricci et al. [18], who observed that fermentation and subsequent storage of elderberry juices, using selected lactic acid bacteria (*L. plantarum, L. rhamnosus,* and *L. casei*) isolated from both vegetable and dairy resources, result in an increment in the concentration of volatile compounds, including ketones especially diacetyl and acetoin. This could be the reason for the highest diacetyl content in the EPY during last 2 weeks of storage.

### 3.2. Microbiology

The viability of the starter bacteria was not influenced by the addition of fruit purees. Moreover, their level during the entire storage period was never below 10^6^ cfu/g, which is particularly important in the case of probiotic cultures, i.e., *Lb. acidophilus* La-5 and *B. animalis* ssp. *lactis* BB-12, because this concentration is generally accepted as the minimum for the final product to show health benefits [19]. Different factors may influence the growth and viability of probiotic cells in yoghurt, such as milk solid content, availability of nutrients, buffering capacity, selection and dosage of starter strains, culture conditions, fermentation time, storage conditions, β-galactosidase concentration, and type of packaging [19,20,21]. Additional factors, which may play a crucial role in fruit yoghurts, include oxygen incorporated during stirring of the yoghurt milk base with fruits, acidity of the added fruit mix, sugar concentration (osmotic pressure), and possible preservatives in fruit preparations. In general, probiotic bacteria, especially bifidobacteria, used in fermented milks have low tolerance toward acids and oxygen. On the other hand, fruits contain dietary fibers with a potential prebiotic effect and many antioxidant components (e.g., polyphenols, vitamins), which may potentially eliminate oxygen from the environment and, thus, enhance probiotic survivability [21]. According to Sun-Waterhouse et al. [22], the effect of bioactive compounds present in fruits on the viability of starter bacteria may be positive (stimulating) or negative (antimicrobial) depending on the type of bioactive ingredients and bacteria type. For example, catechins were reported to have a stimulating effect on lactic acid bacteria, whereas isoflavones and phytosterols negatively influence the count of probiotic cells.

The results of some reports suggested that sea buckthorn berries may improve the viability and serve as an efficient immobilization carrier to protect probiotic cells (e.g., *L. casei* ATCC 393) in dairy matrices [23,24,25]. Contradictory results were obtained by Selvamuthukumaran and Farhath [4] for sea buckthorn fruit yoghurt, as these authors observed a significant decrease in the starter bacteria count during storage. However, there were certain differences between the aforementioned study and our research, the most important being the addition of fruit syrup before fermentation and the application of different cultures, i.e., traditional yoghurt bacteria (*Lb. delbrueckii* ssp. *bulgaricus* and *Str. thermophilus*). Kailasapathy et al. [20] also observed a decrease in bifidobacteria and *Lb. acidophilus* cell viability in stirred fruit ABT yoghurts during storage, but the final count of both species did not drop below the recommended level for probiotics (10^6^–10^7^ cfu/g). Moreover, cited authors suggested that the best pH to ensure the high rate of cell viability is in the range of 4.4–4.5 for bifidobacteria and 4.1–4.5 for *Lb. acidophilus*. Since, in our study, the pH of yoghurts during the entire storage period never decreased below 4.3, this could have contributed to the high level of bacteria viability.

### 3.3. Antioxidant Capacity

According to the literature, the incorporation of various fruit preparations (pulps, juices, etc.) into the yoghurt formula enhances the antioxidant potential of the fermented milk because fruits are an exceptionally rich source of antioxidant substances, such as polyphenols, vitamin C and other vitamins, and carotenoids [26,27]. All the analyzed fruit purees were good sources of polyphenolic compounds, although their polyphenolic profiles differed (Table 1). In the sweetened and pasteurized sea buckthorn berry puree, the main group of polyphenolic substances included (−)-epicatechin and six phenolic acids detected in considerable amounts, with salicylic acid present in the highest concentration. This is in agreement with the literature, as Zadernowski et al. [28] also reported salicylic acid as the main phenolic acid in sea buckthorn fruits. In contrast, elderberry puree contained anthocyanins and rutin as the major polyphenols. The same was stated by Da Silva et al. [8], who identified cyanidin-3-*O*-sambubioside, cyanidin-3-*O*-glucoside, and cyanidin-3-*O*-sambubioside-5-*O*-glucoside as the most abundant anthocyanins in elderberry juice, and the study of Domínguez et al. [10], who reported that rutin and, to a lesser extent, quercetin were the main representatives of the flavonoids group in the elderberries. Cosmulescu et al. [29] identified five flavonoids and 10 phenolic acids in the fruit extract of wild-grown blackthorn. Epicatechin, myricetin, and quercetin were the predominant flavonoids, whereas coumaric, elagic, salicylic, sinapic, and vanillic acids were the dominant phenolic acids in the sloe berries studied by these authors. This is not fully consistent with the findings of the present study, as myricetin was the only detected flavonoid in sloe berry puree, whereas chlorogenic and caffeic acids were the most abundant compounds among phenolic acids. The discrepancies between the results obtained by different authors resulted from a different botanical origin of the plant material. In our case, heat treatment of fruits with the addition of saccharose may have also affected the content of certain phenolic compounds.

As all of these polyphenolic compounds found in fruit purees are known as potent antioxidant substances, it was expected that the added fruit preparations should considerably increase antioxidant capacity of the probiotic yoghurts. Indeed, both elderberry and sloe berries significantly improved the radical-scavenging activity toward the DPPH radical (ARP) and ferric reducing ability (FRAP) of the analyzed fermented milks. One can hypothesize that this resulted from the high content of phenolic compounds, including anthocyanins, known for their excellent antioxidant properties, in elderberry and sloe berry preparations. Although these fruits contained the highest concentrations of both groups of compounds, a strong correlation was found only between the anthocyanin content and FRAP values (*r* = 0.87, *r^2^* = 0.76), whereas, for the other pairs of parameters, correlations were much weaker (*r* ≤ 0.75 and *r^2^* ≤ 0.57). This is consistent with the study of Trigueros et al. [30], who found better correlations between phenolic compounds (TPC and TMAC) and FRAP values (*r* = 0.89 and 0.72, respectively) when compared to the results of DPPH assay (*r* = 0.64 and 0.24, respectively) in pomegranate yoghurt. Moreover, in the present study, FRAP and ARP values were quite stable during storage, whereas the total content of phenolics in both treatments and monomeric anthocyanins in SPY tended to decrease during storage. Contradictory results were reported by Karaaslan et al. [31], for yoghurts fortified with grape and callus extracts, where the reduction observed in phenolic and anthocyanin content during storage was closely related to a decline in antioxidant capacity determined by means of DPPH assay. On the other hand, in the study of Trigueros et al. [30], the anthocyanins determined in pomegranate yoghurts using a spectroscopic method were stable during storage, whereas FRAP values significantly decreased. Surprisingly, the antioxidant properties of SBPY did not differ significantly from the natural one, particularly when the FRAP method was taken into account. On one hand, the content of anthocyanins in sea buckthorn was very low compared to other applied fruits and did not bring any difference when SBPY was compared to the PPY in terms of TMAC; on the other hand, sea buckthorn added great value to yoghurts with respect to TPC, phenolic acids, epicatechin, and myricetin. This could have been due to too small an addition of fruit puree (10% of fruit puree equal to 7% of berries) or the methods used for the evaluation of the antioxidant capacity. This observation is consistent with the results reported by Najgebauer-Lejko and Sady [27] performed on commercial yoghurt samples, which demonstrated that yoghurts with bluish-purple and red berries (bilberry, forest fruit, strawberry, cherry, and blackcurrant) were characterized by higher FRAP and ARP values than the respective yoghurts with light-colored fruits (peach, pineapple, and apricot). High stability of the antioxidant capacity measured using ARP and ORAC methods in bilberry and blackcurrant yoghurts during storage was also reported by Skrede et al. [32]. In the literature [28,33], it was reported that the high antioxidant power of sea buckthorn berries results to a considerable degree from the presence of antioxidant compounds other than polyphenols, e.g., vitamin C and carotenoids. However, the relatively low antioxidant capacity of the SBPY stated in the present research may have been due to the fact that vitamin C is sensitive to the high temperatures applied during the pasteurization of fruit purees. Moreover, carotenoids, another antioxidant substance present in sea buckthorn berries, may react poorly in the conditions used for the ARP assay; therefore, the antioxidant capacity of carotenoid-rich fruit may have been underestimated [32].

### 3.4. Color

The color of yoghurt is an important factor influencing its general appearance and visual attractiveness to consumers, and fruit yoghurt should reflect the color intensity, hue, and shade of the respective fruit. Natural or artificial colorants may be added to yoghurt to achieve this goal, or the color of the final product may result exclusively from the natural fruit color. Currently, there is a great shift of consumers’ and industry preference toward natural colorants and an avoidance of artificial coloring agents, as they are negatively associated with health issues, e.g., with an adverse effect on activity and attention in children [9,34]. Among natural colorants, elderberry juice is commonly used as a source of anthocyanins in dark fruit yoghurts, e.g., bilberry and blueberry, which give a red to bluish-purple color to the fermented milk. In the case of adding coloring substances, pH should be controlled as some of them may have different colors in different acidities of the environment, e.g., anthocyanins are highly prone to pH changes. Moreover, some colorants, e.g., beetroot betaine, are unstable during heat treatment [34]. Anthocyanins, present in considerable quantities in the elderberry and sloe berry purees (Table 1), present a color from red, through purple, to blue depending on the pH of the environment [35]. Therefore, in the acidic environment of probiotic yoghurt, a relatively dark, reddish-purple color of the elderberry and sloe berry yoghurts was expected. However, being highly reactive compounds, anthocyanins are known to be unstable and prone to degradation, resulting in colorless or brown-colored compounds [31]. The lack of significant changes in the levels of color parameters during storage suggests the stability of anthocyanins in these yoghurt treatments. However, this observation is not fully consistent with the results of anthocyanin content. Indeed, TMAC was stable in the EPY but a dramatic drop in anthocyanin concentration was observed in the SPY after 1 month of cold storage. No significant changes in color parameters and TMAC in pomegranate yoghurt were obtained by Trigueros et al. [30], who suggested that milk matrices may have a positive influence on anthocyanin and color stability, together with inter- and intramolecular, co-pigmentation, and self-association reactions. The lack of significant differences in color parameters during storage while observing a decrease in anthocyanin content may have resulted from their condensation with other phenolic compounds (e.g., phenolic acids, flavan-3-ols) to colored polymeric pigments [36]. Moreover, acetaldehyde, which is one of the major aromatic compounds found in yoghurt, is known to accelerate this reaction. Many factors are known to affect anthocyanin stability such as pH, storage temperature, type of bacterial culture, content of fat and other components of food matrix (e.g., ascorbic acid), type (molecular weight, structure) and concentration of phenolic compounds, interactions with proteins, and oxygen [30], which also affect their color. All of the aforementioned parameters may be of high importance in yoghurts fortified with different additives containing anthocyanins, e.g., different fruit species [37].

In contrast to EPY and SPY, SBPY was characterized by a brighter, yellow–orange, and more saturated color which was stable during storage. This resulted from the high concentration of carotenoids, particularly β-carotene, lycopene, and zeaxanthin in sea buckthorn berries [38].

### 3.5. Texture and Syneresis

The following parameters were measured using the back extrusion test (BET): firmness, defined as the force needed to achieve deformation for a given penetration distance; cohesiveness, which indicates the resistance of the sample to withdrawal from the extrusion disc which is lifted; consistency, related to the thickness of the sample; index of viscosity, referring to the resistance of the sample to flow off the disc during the test [39]. According to Sikora et al. [40], sloe berries contain on average 5.79 g (fresh) or 4.79 g (freeze stored) of fiber in 100 g FW. Among different treatments obtained in our study, SPY was characterized by the highest content of dietary fiber. Other fruit purees also significantly increased fiber concentration in yoghurts when compared to PPY. This compound, together with some other polysaccharides naturally occurring in fruits, may have a significant effect on the textural properties of probiotic yoghurts, for example, causing an increase in consistency and viscosity [41]. In most cases, the fruit addition did not affect the textural parameters. However, SPY, when compared to SBPY and EPY, tended to show higher values of firmness, consistency, cohesiveness, and index of viscosity. In the study of Ürkek et al. [12], the addition of sloe berries to ice cream mix resulted in a significant increase in viscosity, probably due to the high dietary fiber content in the fruits. Slightly higher values of the textural parameters in our study were not accompanied by inhibition of the syneresis phenomenon. The lack of significant differences in the textural studies may have resulted from the relatively small addition of fruit preparations (10% of sweetened fruit puree equal to 7% of fruits in yoghurts). However, a preliminary study performed on the optimal level of fruit addition (data not shown) suggested that a higher addition level can result rather in pronounced defects in consistency and excessive syneresis. The structure and textural properties of yoghurt gel are the result of the arrangement of the three-dimensional network formed mainly by milk proteins. The addition of a fruit preparation may disrupt protein matrix integrity and, thus, negatively influence the appearance and textural characteristics of the product. Commercial yoghurts usually contain stabilizers to avoid such problems but we decided not to add any in order to produce products as natural as possible and to avoid an additional variability factor.

### 3.6. Sensory Quality

The high scores received in a sensory hedonic-scale experiment indicate that fruit yoghurts were characterized by acceptable sensory attributes such as color, taste, odor, consistency, and general appearance. Similar results were obtained for the fresh sea buckthorn yoghurt in the study of Selvamuthukumaran and Farhath [4]. However, the authors indicated that yoghurt maintained sensory properties acceptable up to 18 days of cold storage, and a decrease in overall acceptability was caused by increased acidity, off-flavors, and syneresis. It should be mentioned that the yoghurt studied by Selvamuthukumaran and Farhath [4] was produced using different fruit preparations and levels of addition (15% of fruit syrup), with a stabilizer (gelatin), and with different starter cultures (classic yoghurt culture without probiotics) and procedures (fermentation after addition of fruit preparation). In the study of Terpou et al. [25], frozen yoghurt samples supplemented with sea buckthorn berry immobilized probiotic cells received high scores of preference in a sensory evaluation, and the citrus flavor provided by this fruit additive positively influenced total acceptance scores. In our research, the flavor of sea buckthorn was less preferred by the sensory evaluators, probably because it was too intense and the panelists were not familiar with it. In the study of Du and Myracle [35], kefir with the addition of elderberry juice received high scores in a sensory evaluation. The authors emphasized that consumer acceptance was significantly affected by the sugar addition; higher sucrose content led to higher ratings received in a sensory assessment. In our study, we used the same sucrose dosage in all products. A greater addition of sugar, particularly to the most sour sea buckthorn yoghurt, could contribute to an increase in consumer preference.

## 4. Materials and Methods

### 4.1. Materials

Although sea buckthorn grows as a wild tree in selected areas of Poland, gathering fruits from these areas is prohibited as this plant species is protected. Consequently, sea buckthorn berries (*Hippophae rhamnoides* L.) were obtained from the Horticultural Farm Stanisław Trzonkowski from Sokółka (Podlaskie voivodeship, Poland). Elderberries (*Sambucus nigra* L.) and sloe berries (*Prunus spinosa* L.) were gathered manually in the wild near the village of Wygiełzów located in the Polish Jurassic Highland in the south part of Poland. Elderberries were collected at full maturity at the end of September, whereas sloe berries were collected after the first frost in October when fruits lose their astringency. Only fresh undamaged fruits, of proper size and color, were selected, washed out, dried at room temperature, frozen, and stored in plastic bags at −18 °C prior to puree preparation.

Yoghurts were produced from Holstein-Friesian cows’ milk obtained from a local milk farm KHNO “Polan sp. z o.o.” in Dziekanowice (Poland).

Skim milk powder (35.7% of protein) came from the Dairy Cooperative in Gostyń (Poland).

Probiotic, lyophilized ABT-1 culture (Chr. Hansen, Hoersholm, Denmark), composed of the following bacterial strains, was used for milk fermentation: *Lactobacillus acidophilus* La-5, *Bifidobacterium animalis* ssp. *lactis* BB-12, and *Streptococcus thermophilus* (product datasheet, Chr Hansen). The culture was of the DVS (for direct milk inoculation) type.

M17 and MRS agars and peptone water were purchased from Biocorp (Warszawa, Poland). Folin–Ciocalteu’s phenol reagent, Trolox ((±)-6-hydroxy-2,5,7,8-tetramethylchromane-2-carboxylic acid), and gallic acid monohydrate were purchased from Fluka (Buchs, Switzerland; Copenhagen, Denmark and Madrid, Spain), whereas 2,2-diphenyl-1-picrylhydrazyl (DPPH) and 2,4,6-tris(2-pyridyl)-*s*-triazine (TPTZ) were purchased from Sigma–Aldrich (Steinheim, Germany and Buchs, Switzerland). The following HPLC-grade standards were used for HPLC analysis: caffeic acid, vanillic acid, protochateuchic acid, ferulic acid, rutin, kaempferol, (+)-catechin, (−)-epicatechin (Sigma Aldrich, China), salicylic acid (Chempur, Poland), *p*-coumaric acid, ellagic acid (Sigma Aldrich, UK), quercetin (Sigma Aldrich, India), chlorogenic acid (Sigma Aldrich, Switzerland), gallic acid, and 4-hydroxy-benzoic acid (Merck, Germany). All other chemicals used were of analytical reagent grade.

### 4.2. Preparation of Fruit Purees

Before preparation, the fruits were thawed at ambient temperature for about 1 h. Subsequently, they were placed over a boiling water bath for 15 min (but boiling of juice was avoided), manually blended, and rubbed through a sieve with a spoon to remove solid parts. After addition of sucrose in the amount of 30 g per 100 g of fruits, purees were placed in 200 mL glass jars and pasteurized in a water bath at 85 °C for 15 min. After cooling in cold water, the purees were stored at 4 °C before addition to probiotic yoghurts.

### 4.3. Production of Natural and Fruit Probiotic Yoghurts

**Figure 3:**
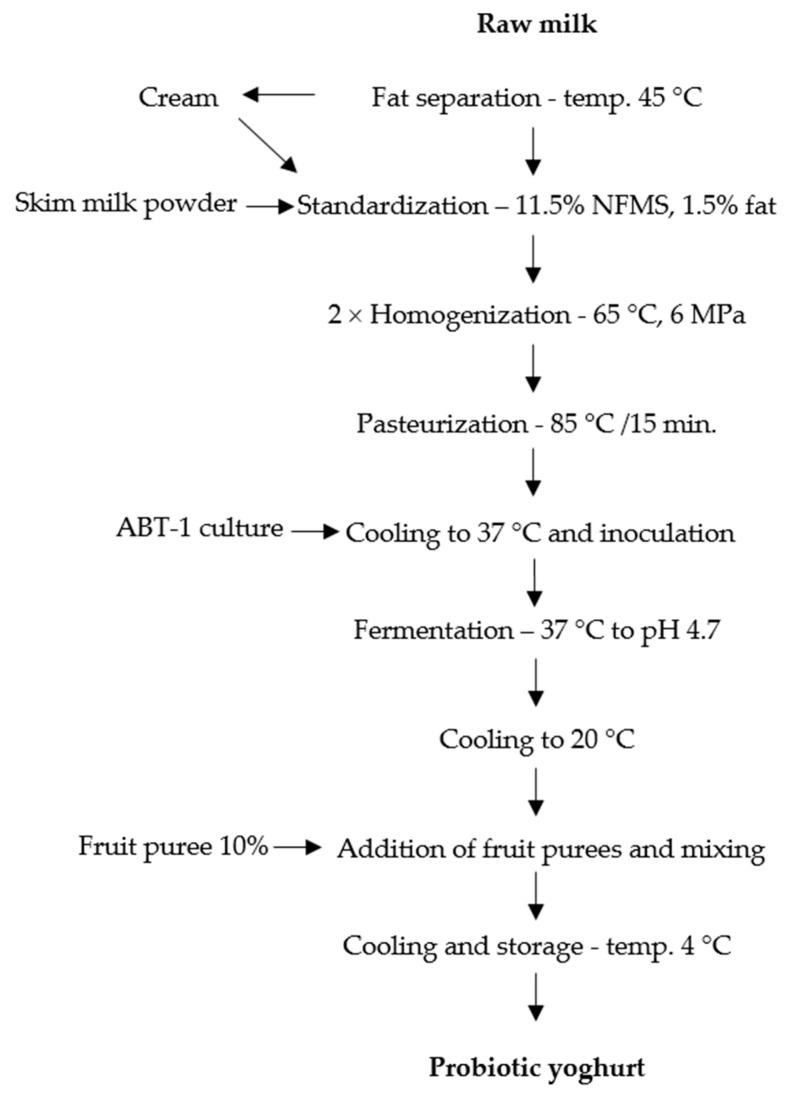
Fruit yoghurt production scheme.

Figure 3 presents the production steps of probiotic yoghurts. Fresh cow milk was heated to 45 °C and centrifuged (LWG24E milk separator—Spomasz, Gniezno, Poland) to separate cream from skim milk. Fat content was standardized to 1.5% by mixing of skim milk with cream in proper proportions, and the non-fat solids were standardized to 11.5% content by the addition of skim milk powder to warm (50 °C) milk. After standardization, the milk was heated to 65 °C prior to twofold homogenization (FT-9 Armfield milk homogenizer, Ringwood, England) at 6 MPa and pasteurized in a water bath at 85 °C for 15 min. After cooling to 37 °C, the milk was inoculated with the ABT-1 culture (0.075 g/L), poured into 200 mL sterile glass jars, and incubated at 37 °C until a pH of 4.7 was reached (10–12 h). After incubation, the yoghurts were cooled to the temperature of about 20 °C. At this temperature the respective fruit puree was added in the amount of 10 g/100 g and mixed manually with a sterile spoon for 5 min to obtain a uniform color. One-fourth of the yoghurts in jars was left without addition but mixed in the same way as the fruit treatments. Finally, the yoghurts were cooled to 4 °C and stored at this temperature in a refrigerator until analyses were performed.

The following treatments were produced:

- Natural (plain) probiotic yoghurt without any fruit additive (NPY);
- Probiotic yoghurt with 10% sea buckthorn fruit puree (SBPY);
- Probiotic yoghurt with 10% elderberry puree (EPY);
- Probiotic yoghurt with 10% sloe fruit puree (SPY).

### 4.4. Methods

#### 4.4.1. Chemical Composition

The concentrations of the following components were determined: total solids by drying at 130 °C, fat by the gravimetric Gerber’s method, protein by the Kjeldahl method using the KjelFlex K-360 Büchi apparatus (Büchi Labortechnik AG, Flawil, Switzerland), total carbohydrates by the Bertrand’s method, and ash content by dry incineration in a muffle furnace at 550 °C [42]. Total dietary fiber content was evaluated by the enzymatic–gravimetric method [43] using the Megazyme total dietary fiber assay kit (Megazyme Int., Bray, Ireland).

#### 4.4.2. Acidity

To determine titratable acidity, 25 mL of the yoghurt sample was diluted with 25 mL of distilled water and titrated with 0.25 N NaOH to a light pink using a 2% ethanolic solution of phenolphthalein as an indicator of the end point (for plain, white yoghurt) or using a pH meter to reach a pH value of 8.3 (for fruit, colorful yoghurts). Titratable acidity was calculated per 100 mL and expressed as percentage lactic acid using a conversion rate of 0.0225 [44]. Additionally, pH was measured in the yoghurt samples using an Elmerton CP-411 pH-meter (Elmetron Sp.j., Zabrze, Poland).

#### 4.4.3. Antioxidant Capacity

Antioxidant properties of the probiotic yoghurts were evaluated as ferric reducing antioxidant potential (FRAP) and ability to scavenge DPPH radicals (ARP—antiradical power). Both spectroscopic methods were described in detail by Najgebauer-Lejko et al. [45]. Results of the FRAP analysis were expressed as mM Fe^2+^/kg, whereas ARP was expressed as mM TE (Trolox equivalent)/kg of the sample.

#### 4.4.4. Total Phenolic Content (TPC)

Extracts for TPC assay were obtained by mixing 10 g of the sample in 10 mL of 60% ethanol solution using a vortex for 30 min at ambient temperature. The mixture was centrifuged for 15 min at 2683× g, and the clear supernatant was used for analysis. TPC in probiotic yoghurt samples was determined by the Folin–Ciocalteu method. In this method, 0.1 mL of yoghurt extract after centrifugation was mixed with 7.9 mL of distilled water and 0.5 mL of 2 N Folin–Ciocalteu reagent. Exactly after 30 s, 1.5 mL of a 20% aqueous solution of sodium carbonate was added, and the whole solution was thoroughly shaken. The mixture was left for 2 h at room temperature (~20 °C) to react. The absorbance of the reaction liquid was measured at 765 nm using a UV–Vis Helios Gamma spectrophotometer (Thermo Electron Corp., Cambridge, UK) and the results were read from a calibration curve and expressed as mg GAE (gallic acid equivalents) per 100 g of the probiotic yoghurt sample.

#### 4.4.5. Total Monomeric Anthocyanin Content (TMAC)

TMA content was determined by the pH-differential spectroscopic method reported by Karaaslan et al. [31], Lee et al. [46], and Wrolstad et al. [36] with some modifications. Briefly, 10 g of yoghurt or puree sample was mixed with 15 mL of acidified methanol (1 mL concentrated HCl per 100 mL of methanol, pH 2.0) and left at 4 °C overnight. Subsequently, the mixture was transferred to centrifugation tubes, adjusting the volume to 50 mL with acidified methanol, and centrifuged for 10 min at 2683× *g* using MPW 352R centrifuge (MPW Med. Instruments, Warsaw, Poland). Methanolic extracts of probiotic yoghurts (supernatants) were pipetted in the amount of 1 mL to 20 mL (yoghurts) or 200 mL (fruit purees) of the proper buffer solution and thoroughly mixed. Each sample was diluted in two buffer solutions, i.e., 0.025 M potassium chloride, pH 1.0 and 0.4 M sodium acetate, pH 4.5. The absorbance was read for each sample at two wavelengths, i.e., 520 nm and 700 nm and at two pH values (1.0 and 4.5) using a UV–Vis Helios Gamma spectrophotometer (Thermo Electron Corp., Cambridge, UK). Total monomeric anthocyanin content (TMAC) was calculated from the following equation:

(1) $$TMAC=\frac{A\;\cdot \;MW\;\cdot \;DF\;\cdot \;10^{3}}{Ɛ\;\cdot \;l},$$

where A is the absorbance (= (A_520_ − A_700_)_pH 1,0_ − (A_520_ − A_700_)_pH 4,5_, MW is the molecular weight (= 449.2 g/M for cyanidin-3-glucoside), DF is the dilution factor, 10^3^ is the conversion factor from g to mg, l is the pathlength (1 cm), and Ɛ is the molar extinction coefficient (= 26,900 L·M^−1^·cm^−1^ for cyanidin-3-glucoside). The results were expressed as mg CGE (cyanidin-3-glucoside equivalents) per 100 g of probiotic yoghurt sample.

#### 4.4.6. HPLC Analysis of Phenolic Acids and Flavonoids in Fruit Purees

The samples of fruit purees for HPLC assay were prepared according to the procedure described by Klimczak et al. [47] with some modifications. Lyophilized fruit purees (~0.1 g) were dissolved in 1% methanolic (HPLC grade) l-ascorbic acid solution, mixed using a vortex mixer Labnet (Edison, NJ, USA) and sonicated for 30 min at 20 °C using an IS-14 ultrasonic bath (InterSonic, Olsztyn, Poland). The tightly closed mixtures were cooled and stored at 4 °C for 12 h. Next, the mixtures were hydrolyzed in 2 M NaOH added in a proportion of 1:1 (*v*/*v*). After mixing with a vortex, the mixtures were left in darkness at ambient temperature for 4 h. Subsequently, the mixtures were neutralized to pH 2.1–2.6 with 2 M HCl, centrifuged at 5000 rpm for 10 min at 4 °C (352 RH centrifuge, MPM, Warszawa, Poland), and adjusted to the volume of 5 mL with 1% methanolic l-ascorbic acid solution. Prior to the HPLC assay, mixtures were centrifuged at 18,000 rpm for 20 min at 4 °C (260-R centrifuge, Warszawa, Poland), and supernatants were filtered through PTFE-L 22 µm filters were stored at 4 °C. The HPLC assay was performed using an HPLC Dionex UltiMate 3000 chromatograph equipped with a DAD Thermo Scientific (Thermo Fisher Scientific, Germering, Germany) detector and Cosmosil 5C_18_-MS-II (250 × 4.6 mm ID, 5 µm) column (Nacalai Tesque, INC. Kyoto, Japan). The mobile phase consisted of two solvents: solvent A, 2% acetic acid aqueous solution; solvent B, 100% methanol. The flow rate was set as 1 mL/min. The whole analysis was performed according to the following arrangement: solvent A 70% for 10 min, 50% for the next 15 min, 30% for the next 10 min, 95% for the next 15 min, and ≥ 95% until the end of the assay. The contents of phenolic and carboxylic acids, as well as flavonoids (flavonols, flavonol glycosides, and catechins), were identified and quantified using the corresponding standards at the following wavelengths: 245 nm, 280 nm, 320 nm, and 360 nm.

#### 4.4.7. Microbiological Analyses

Prior to analysis, decimal dilutions of yoghurt samples were prepared in peptone water. The number of *Streptococcus thermophilus* colonies was evaluated applying the pour-plate technique using M17 agar (6.8 pH) under aerobic conditions [48]. *Lactobacillus acidophilus* colonies were enumerated using MRS-maltose agar (pH 6.4) prepared from the same components and procedure as MRS agar with the exception of substituting glucose with an equal amount of maltose [49]. The levels of bifidobacteria were grown on NNLP-MRS agar, i.e., MRS with 5% NNLP supplement (nalidixic acid, neomycin sulfate, lithium chloride, and paromomycin sulfate) [50]. Streptococci and lactobacilli were incubated under aerobic conditions, whereas bifidobacteria were grown anaerobically in CO_2_ incubators of our own construction. All cultures were incubated at 37 °C for 48 h (streptococci) or 72 h (lactobacilli and bifidobacteria). Samples of probiotic yoghurts were also checked for contamination with yeast and molds using chloramphenicol agar at 25 °C for 5 days [51].

#### 4.4.8. Acetaldehyde and Diacetyl Contents in Probiotic Yoghurts

Acetaldehyde content was determined in yoghurts using the Conway micro-diffusion method [52]. At first, the following reagents were added to the inner (center) well of the Conway micro-diffusion unit: 2.5 mL of 0.2% 3-methyl-2-benzothiazolinone hydrazine hydrochloride and 0.25 mL of dimethyl sulfoxide. Then, 2 mL of 0.66 N sulfuric acid, 2 mL of sodium tungstate, and 2 mL of the probiotic yoghurt sample (or distilled water in case of blind sample) were pipetted into the outer well of the micro-diffusion unit. The mixtures were gently mixed with a glass rod separately in the inner and outer wells. The whole unit was tightly covered with a glass cover and incubated at 30 °C for 90 min. After incubation, mixtures were cooled to room temperature for up to 15 min. Subsequently, 2 mL of mixture from the inner chamber was transferred into a conical flask and 5 mL of 0.2% ferric chloride in hydrochloric acid was added and mixed. After 25 min, 10 mL of acetone was added, and the absorbance was measured immediately at 660 nm against a blank using a UV–Vis Helios Gamma spectrophotometer (Thermo Electron Corp., Cambridge, UK). The results were read from a calibration curve and expressed as mg acetaldehyde per 100 mL of the yoghurt sample.

Diacetyl content was estimated by the steam distillation method according to Pien [53]. Briefly, 100 g of yoghurt was weighed out into the distillation bulb flask and distilled using a glass apparatus to obtain 20 mL of distillate. Subsequently, 0.75 mL of freshly prepared 2.5% 3,3”-diaminobenzidine tetrahydrochloride hydrate aqueous solution and 0.75 mL of concentrated HCl (d = 1.19 g/mL) were added to 20 mL of distillate; after mixing, the absorbance was measured using a UV–Vis Helios Gamma spectrophotometer (Thermo Electron Corp., Cambridge, UK) at 436 nm against a blank sample prepared with distilled water. The results were read from the calibration curve and expressed in mg per 100 g of probiotic yoghurt sample.

#### 4.4.9. Textural Studies

The back extrusion test (BET) was employed to study textural properties of the natural and fruit probiotic yoghurts. BET was performed using a TA-XTPlus texture analyzer (Stable Micro Systems, Haslemere, Surley, UK) on the undisturbed yoghurt samples in original glass jars (inner diameter of 55 mm, sample height of 60 mm) removed from a refrigerator (4 °C) directly before analysis. The test was performed using a plastic cylindrical probe (diameter: 50 mm, height: 5 mm), which was thrust through the sample to a set depth of 30 mm at a speed of 1 mm/s. On the basis of the force vs. time function, the following parameters were calculated using the appropriate software: firmness (maximum force of the extrusion; N), cohesiveness (minimum force of the extrusion; ∣N∣), consistency (positive area of the force vs. time curve, N∙s), and index of viscosity (negative area of the force vs. time curve; ∣N∙s∣).

#### 4.4.10. Susceptibility to Syneresis

The centrifugal method was employed to measure susceptibility of yoghurt samples to syneresis. Graduated plastic tubes were gently filled with yoghurt samples to the volume of 10 mL using a spoon and centrifuged at 800× *g* for 10 min. The volume of expelled whey was read from the scale and calculated as per 100 mL of yoghurt sample (% vol).

#### 4.4.11. Color Profile

Color profiles of yoghurt samples were measured using a Konica Minolta CM-3500 d spectrophotometer (Konica Minolta Sensing Inc., Osaka, Japan) in reflectance mode, using standard illuminant and D65/10°. The following parameters in the CieL*a*b* system were determined: L*—lightness (from 0—absolute black to 100—absolute white), a* coordinate—green (negative values) to red (positive values) color, and b* coordinate—blue (negative values) to yellow (positive values). Additionally, C*—chroma (saturation) and h°—hue angle were calculated. Each sample before measurement was heated to 20 °C and thoroughly mixed.

#### 4.4.12. Sensory Analysis

Sensory evaluation of natural and fruit yoghurts was performed using a five-point hedonic scale (from 1—very bad to 5—excellent, with possible half-notes) by a trained panel of 12 judges chosen from academic staff and students (age of 24–50 years). The yoghurt samples (~50 mL) were served in random order, in plastic, transparent cups coded with three-digit numbers. The following properties of the yoghurts were assessed: color, taste, odor, consistency, and general appearance. The overall preference was calculated taking into account the proper indices of importance for each quality attributes (0.10, 0.35, 0.15, 0.25, and 0.15, respectively).

### 4.5. Experimental Design and Statistical Analysis

Probiotic yoghurts were produced in two independent series (production procedures were repeated twice), and all analyses were performed in two replicates (*n* = 4). Analyses of the chemical compositions were performed once at the beginning of the study, whereas other analyses were done on the first, 15th, and 28th days of cold storage (4 °C). The results were expressed as means ± SD. The results were subjected to the ANOVA with the use of Statistica 13.3 software (StatSoft, Inc., Tulsa, OK, USA). Type of yoghurt and storage time were the variability factors taken into account and, where applicable, the significance of differences between the average values was estimated on the basis of Tukey’s HSD test at the significance level of *p* ≤ 0.05.

## 5. Conclusions

The addition of elderberry and sloe berries significantly increased the antioxidant capacity of probiotic yoghurts, probably due to a high content of polyphenols, especially anthocyanins. However, anthocyanins were more stable in the EPY when compared to the SPY. Therefore, elderberries have greater potential in terms of adding a dark purple color to yoghurts. In contrast, sloe berry puree may serve as an exceptionally good source of dietary fiber. Sea buckthorn berries did not bring much in terms of antioxidant capacity to the yoghurts but provided a bright yellow color. None of the fruit additives affected the viability of starter microorganisms, including *Lactobacillus acidophilus* La-5 and *Bifidobacterium animalis* ssp. *lactis* BB-12, during 4 weeks of cold storage. Elderberries promoted the evolution of diacetyl in yoghurts during storage and, together with sloe berries, resulted in increased syneresis and the greatest changes in color profile compared to PPY. Summarizing, the obtained results show that purees from elderberries, sea buckthorn, and sloe berries can be successfully used as functional additives to probiotic yoghurt, playing the role of natural colorants and flavorings, as well as being a source of antioxidants and dietetic fiber. However, optimization of the yoghurt formula, especially sucrose content, is recommended taking into account the different acidity of fruits in order to increase the sensory acceptability of the final products.

## Figures and Tables

**Table 1:** The content of polyphenolic compounds in fruit purees (mean ± SD, *n* = 4).

Phenolic Compound	Type of Fruit Puree		
	Sea Buckthorn Berry	Elderberry	Sloe Berry
TPC (mg GAE/100 g DW)	740.59 ± 0.38 ^a^	1617.03 ± 2.44 ^c^	1065.26 ± 22.78 ^b^
TMAC (mg CGE/100 g DW)	81.65 ± 3.78 ^a^	301.98 ± 1.81 ^b^	312.03 ± 44.31 ^b^
Caffeic acid (mg/100 g DW)	325.75 ± 3.07 ^c^	11.34 ± 0.39 ^a^	100.39 ±1.88 ^b^
*p*-Coumaric acid (mg/100 g DW) Salicylic acid (mg/100 g DW)	65.45 ± 2.33 ^c^ 740.64 ± 26.88	2.22 ± 0.23^a^ nd	7.18 ± 0.09 ^b^ nd
Ferulic acid (mg/100 g DW)	306.55 ± 15.99 ^b^	nd	4.11 ± 0.10 ^a^
Chlorogenic acid (mg/100 g DW)	450.82 ± 16.59 ^b^	nd	144.98 ± 1.32 ^a^
*t*-Cinnamic acid (mg/100 g DW)	51.28 ± 1.58	nd	nd
Myricetin (mg/100 g DW)	51.08 ± 3.42 ^b^	nd	14.39 ± 0.11 ^a^
Quercetin (mg/100 g DW)	nd	1.42 ± 0.04	nd
Rutin (mg/100 g DW)	nd	98.77 ± 3.66	nd
(−)-Epicatechin (mg /100 g DW)	1237.13 ± 48.00	nd	nd
(+)-Catechin (mg/100 g DW)	nd	176.06 ± 2.64	nd

Legend: TPC—total phenolic content, TMAC—total monomeric anthocyanin content, CGE—cyanidin-3-glucoside equivalent, GAE—gallic acid equivalent, nd—not detected; statistically significant differences between means at *p* ≤ 0.05 are denoted with different superscript letters.

**Table 2:** Chemical composition of probiotic yoghurts (g/100 g, mean ± SD, *n* = 4).

Component	Yoghurt Type			
	Natural	Sea Buckthorn Fruit	Elderberry	Sloe Berry
Total solids	14.07 ± 0.72 ^a^	16.08 ± 0.51 ^b^	16.14 ± 0.55 ^b^	16.08 ± 1.45 ^b^
Protein	4.61 ± 0.18 ^a^	4.23 ± 0.19 ^a^	4.38 ± 0.57 ^a^	4.13 ± 0.43 ^a^
Fat	1.59 ± 0.11 ^b^	1.63 ± 0.11 ^b^	1.58 ± 0.15 ^b^	1.28 ± 0.12 ^a^
Carbohydrates	6.37 ± 0.31 ^a^	9.22 ± 0.17 ^b^	9.13 ± 0.40 ^b^	9.64 ± 0.16 ^b^
Fiber	0.18 ± 0.00 ^a^	0.40 ± 0.00 ^b^	0.42 ± 0.00 ^c^	1.15 ± 0.00 ^d^
Ash	1.08 ± 0.02 ^c^	0.98 ± 0.02 ^a^	1.04 ± 0.02 ^b^	1.03 ± 0.02 ^b^

Legend: statistically significant differences between means at *p* ≤ 0.05 are denoted with different superscript letters.

**Table 3:** Antioxidant parameters, acidity, number of starter bacteria, and concentrations of aromatic compounds in the probiotic yoghurts during storage (mean ± SD, *n* = 4).

Parameter	Storage Time (Day)	Natural Yoghurt	Sea Buckthorn Fruit Yoghurt	Elderberry Yoghurt	Sloe Berry Yoghurt
TPC (mg GAE/100 g)	1	0.00 ± 0.00 ^a^	636.35 ± 0.21 ^c^	998.40 ± 5.37 ^i^	871.75 ± 1.34 ^g^
	15	0.00 ± 0.00 ^a^	545.25 ± 13.79 ^b^	908.00 ± 0.42 ^h^	790.55 ± 0.77 ^f^
	29	0.18 ± 0.18 ^a^	558.80 ± 5.66 ^b^	724.70 ± 5.51 ^e^	685.00 ± 0.84 ^d^
TMAC (mg CGE/100 g)	1	0.15 ± 0.14 ^a^	2.34 ± 1.45 ^ab^	13.37 ± 1.88 ^c^	9.96 ± 0.00 ^cd^
	15	0.06 ± 0.08 ^a^	2.26 ± 0.11 ^ab^	14.13 ± 2.42 ^c^	8.56 ± 1.06 ^de^
	29	0.00 ± 0.00 ^a^	2.15 ± 0.22 ^ab^	12.19 ± 1.46 ^cd^	4.77 ± 0.41 ^be^
FRAP (mMFe^2+^/kg)	1	1.82 ± 0.67 ^a^	1.73 ± 0.23 ^a^	7.77 ± 1.65 ^b^	7.45 ± 2.72 ^b^
	15	2.61 ± 0.76 ^a^	2.07 ± 0.67 ^a^	9.33 ± 2.17 ^b^	8.26 ± 1.32 ^b^
	29	2.06 ± 1.34 ^a^	2.01 ± 0.56 ^a^	9.33 ± 2.14 ^b^	7.83 ± 2.30 ^b^
ARP (mMTE/kg)	1	0.34 ± 0.14 ^a^	0.78 ± 0.67 ^a^	3.98 ± 1.31 ^b^	6.85 ± 2.15 ^d^
	15	0.37 ± 0.32 ^a^	0.74 ± 0.48 ^a^	3.48 ± 0.69 ^b^	4.92 ± 0.86 ^bc^
	29	0.34 ± 0.15 ^a^	0.72 ± 0.32 ^a^	3.20 ± 0.45 ^b^	6.10 ± 1.25 ^cd^
Titratable acidity (% lactic acid)	1	0.81 ± 0.18 ^a^	1.15 ± 0.06 ^c^	0.95 ± 0.02 ^ab^	1.11 ± 0.07 ^bc^
	15	0.97 ± 0.10 ^abc^	1.15 ± 0.08 ^bc^	1.02 ± 0.01 ^bc^	1.06 ± 0.12 ^bc^
	29	1.06 ± 0.06 ^bc^	1.12 ± 0.03 ^bc^	1.02 ± 0.06 ^bc^	1.10 ± 0.06 ^bc^
pH	1	4.69 ± 0.07 ^c^	4.34 ± 0.08 ^a^	4.64 ± 0.07 ^bc^	4.47 ± 0.09 ^abc^
	15	4.54 ± 0.07 ^abc^	4.32 ± 0.07 ^a^	4.56 ± 0.08 ^abc^	4.44 ± 0.09 ^abc^
	29	4.44 ± 0.11 ^a^	4.31 ± 0.11 ^a^	4.41 ± 0.11 ^abc^	4.38 ± 0.18 ^ab^
*Lb. acidophilus * (log cfu/g)	1	7.70 ± 0.43 ^a^	7.71 ± 0.49 ^a^	7.65 ± 0.40 ^a^	7.70 ± 0.40 ^a^
	15	8.07 ± 0.33 ^a^	7.68 ± 0.56 ^a^	7.86 ± 0.43 ^a^	7.75 ± 0.53 ^a^
	29	7.62 ± 0.82 ^a^	7.70 ± 0.40 ^a^	7.89 ± 0.51 ^a^	8.01 ± 0.34 ^a^
*Bifidobacterium animalis* ssp. *lactis* (log cfu/g)	1	6.32 ± 0.24 ^a^	6.37 ± 0.22 ^a^	6.32 ± 0.56 ^a^	6.32 ± 0.38 ^a^
	15	6.44 ± 0.50 ^a^	6.35 ± 0.50 ^a^	6.35 ± 0.45 ^a^	6.32 ± 0.44 ^a^
	29	6.05 ± 0.19 ^a^	6.47 ± 0.46 ^a^	6.31 ± 0.44 ^a^	6.37 ± 0.42 ^a^
*Str. thermophillus* (log cfu/g)	1	8.22 ± 0.49 ^a^	8.20 ± 0.15 ^a^	8.10 ± 0.31 ^a^	8.45 ± 0.25 ^a^
	15	8.43 ± 0.25 ^a^	8.38 ± 0.40 ^a^	8.17 ± 0.11 ^a^	8.32 ± 0.27 ^a^
	29	8.31 ± 0.05 ^a^	8.52 ± 0.52 ^a^	8.33 ± 0.31 ^a^	8.47 ± 0.50 ^a^
Diacetyl (mg/100 g)	1	2.44 ± 1.28 ^a^	3.91 ± 0.73 ^ab^	4.52 ± 2.43 ^ab^	3.45 ± 1.67 ^ab^
	15	4.19 ± 1.39 ^ab^	5.06 ± 0.99 ^abc^	7.80 ± 0.22 ^c^	5.72 ± 0.19 ^bc^
	29	3.51 ± 1.21 ^ab^	5.37 ± 1.51 ^abc^	7.77 ± 1.14 ^c^	5.75 ± 0.92 ^bc^
Acetaldehyde (mg/100 mL)	1	3.83 ± 0.18 ^abc^	4.51 ± 0.29 ^abc^	3.16 ± 0.63 ^ab^	2.38 ± 0.66 ^a^
	15	4.36 ± 1.75 ^abc^	6.73 ± 0.76 ^c^	4.61 ± 1.45 ^abc^	3.29 ± 2.05 ^ab^
	29	4.72 ± 1.61 ^abc^	5.73 ± 0.60 ^bc^	4.23 ± 0.23 ^abc^	5.98 ± 2.36 ^bc^

Legend: TPC—total phenolic content, TMAC—total monomeric anthocyanin content, FRAP—ferric reducing antioxidant power, ARP—antiradical power, TE—Trolox equivalent, CGE—cyanidin-3-glucoside equivalent, GAE—gallic acid equivalent; different superscript letters denote statistically significant differences between average values within a given parameter at *p* ≤ 0.05.

**Table 4:** Textural and color parameters and syneresis of the probiotic yoghurts (mean ± SD, *n* = 4).

Parameter	Storage Time (Day)	Natural Yoghurt	Sea Buckthorn Fruit Yoghurt	Elderberry Yoghurt	Sloe Berry Yoghurt
Firmness (N)	1	1.60 ± 0.04 ^a^	1.26 ± 0.15 ^a^	1.21 ± 0.36 ^a^	1.67 ± 0.01 ^a^
	15	2.03 ± 0.76 ^a^	1.35 ± 0.20 ^a^	1.39 ± 0.12 ^a^	1.95 ± 0.29 ^a^
	29	2.14 ± 0.16 ^a^	1.52 ± 0.02 ^a^	1.45 ± 0.12 ^a^	1.90 ± 0.33 ^a^
Consistency (N⋅s)	1	40.95 ± 0.56 ^a^	31.99 ± 3.05 ^a^	30.73 ± 10.10 ^a^	43.04 ± 0.16 ^a^
	15	49.00 ± 15.13 ^a^	34.49 ± 6.02 ^a^	36.24 ± 2.39 ^a^	50.70 ± 6.75 ^a^
	29	54.43 ± 3.82 ^a^	40.65 ± 0.73 ^a^	37.44 ± 5.00 ^a^	49.90 ± 8.35 ^a^
Cohesiveness (∣N∣)	1	2.50 ± 0.20 ^bc^	1.52 ± 0.12 ^ab^	1.46 ± 0.58 ^ab^	1.78 ± 0.03 ^abc^
	15	2.16 ± 0.46 ^abc^	1.32 ± 0.23 ^a^	1.50 ± 0.19 ^ab^	2.09 ± 0.31 ^abc^
	29	2.71 ± 0.27 ^c^	1.38 ± 0.00 ^ab^	1.45 ± 0.19 ^ab^	1.61 ± 0.28 ^abc^
Index of viscosity (∣N⋅s∣)	1	6.46 ± 0.05 ^a^	4.50 ± 0.35 ^a^	4.23 ± 1.62 ^a^	5.28 ± 0.21 ^a^
	15	5.57 ± 1.08 ^a^	3.72 ± 0.74 ^a^	4.28 ± 0.35 ^a^	5.61 ± 0.73 ^a^
	29	6.39 ± 0.01 ^a^	3.83 ± 0.26 ^a^	4.14 ± 0.54 ^a^	4.45 ± 0.77 ^a^
Syneresis (% vol)	1	14.40 ± 5.73 ^a^	19.28 ± 1.80 ^ab^	29.25 ± 3.72 ^b^	25.77 ± 4.32 ^b^
	15	19.47 ± 3.44 ^ab^	21.13 ± 1.06 ^ab^	23.23 ± 4.63 ^ab^	20.58 ± 1.99 ^ab^
	29	21.52 ± 10.40 ^ab^	21.02 ± 5.54 ^ab^	28.50 ± 2.87 ^b^	24.08 ± 8.15 ^ab^
L*	1	93.49 ± 0.25 ^d^	87.20 ± 1.31 ^c^	57.94 ± 0.21 ^a^	65.75 ± 3.24 ^b^
	15	93.20 ± 0.15 ^d^	87.02 ± 0.29 ^c^	57.46 ± 0.65 ^a^	65.12 ± 2.16 ^b^
	29	93.60 ± 0.50 ^d^	87.02 ± 0.46 ^c^	57.59 ± 0.38 ^a^	65.33 ± 2.61 ^b^
a*	1	−2.22 ± 0.06 ^a^	5.73 ± 0.82 ^b^	15.83 ± 1.25 ^cd^	13.74 ± 2.67 ^c^
	15	−2.15 ± 0.20 ^a^	5.69 ± 0.34 ^b^	16.26 ± 2.07 ^cd^	15.63 ± 3.78 ^cd^
	29	−2.07 ± 0.13 ^a^	5.87 ± 0.30 ^b^	16.88 ± 1.74 ^d^	15.16 ± 3.83 ^cd^
b*	1	10.95 ± 0.21 ^b^	26.48 ± 2.86 ^c^	−2.35 ± 0.51 ^a^	−2.02 ± 2.44 ^a^
	15	11.14 ± 0.04 ^b^	26.04 ± 0.86 ^c^	−1.84 ± 0.53 ^a^	−2.63 ± 2.88 ^a^
	29	11.36 ± 0.03 ^b^	26.13 ± 0.49 ^c^	−1.66 ± 0.43 ^a^	−2.63 ± 2.97 ^a^
*h*	1	101.49 ± 0.31 ^b^	77.83 ± 0.52 ^a^	351.62 ± 1.19 ^c^	353.01 ± 7.56 ^c^
	15	100.57 ± 0.82 ^b^	77.65 ± 1.08 ^a^	353.64 ± 1.05 ^c^	351.24 ± 4.88 ^c^
	29	100.32 ± 0.67 ^b^	77.33 ± 0.76 ^a^	354.46 ± 0.91 ^c^	351.10 ± 4.50 ^c^
*C*	1	11.18 ± 0.22 ^a^	27.10 ± 2.97 ^c^	16.00 ± 1.31 ^b^	14.00 ± 3.05 ^ab^
	15	11.34 ± 0.07 ^a^	26.66 ± 0.78 ^c^	16.37 ± 2.12 ^b^	15.93 ± 2.09 ^b^
	29	11.55 ± 0.01 ^a^	26.79 ± 0.45 ^c^	16.96 ± 1.77 ^b^	15.44 ± 2.56 ^b^

Legend: different superscript letters denote statistically significant differences between average values within a given parameter at *p* ≤ 0.05.

## Data Availability

The data presented in this study are available on request from the corresponding author.

## Sample Availability

Samples of the compounds are not available from the authors.

## References

[1] Aryana K.J., Olson D.W. (2017). A 100-year review: Yoghurt and other cultured dairy products. J. Dairy Sci..

[2] Tamime A.Y., Robinson R.K. (1999). Yoghurt Science and Technology.

[3] O’Rell K.R., Chandan R.C., Chandan R.C., White C.H., Kilara A., Hui Y.H. (2006). Yogurt: Fruit preparations and flavoring materials. Manufacturing Yogurt and Fermented Milks.

[4] Selvamuthukumaran M., Farhath K. (2014). Evaluation of shelf stability of antioxidant rich seabuckthorn fruit yoghurt. Int. Food Res. J..

[5] Selvamuthukumaran M., Khanum F. (2015). Optimization of seabuckthorn fruit yogurt formulation using response surface methodology. J. Food Sci. Technol..

[6] Sidor A.M., Gutt G., Dabija A., Sanduleac E.T., Sidor V. (2017). The effect of yogurt enrichment with sea buckthorn powder on its sensory acceptance, rheological, textural and physicochemical properties. International Multidisciplinary Scientific GeoConference.

[7] Tifrea A., Tiţa O., Máthé E., Ketney O. (2013). Physicochemical parameters of probiotic yoghurt with bioactive natural products from sea buckthorn. Acta Univ. Cibiniensis Ser. E Food Technol..

[8] Da Silva R.F., Barreira J., Heleno S.A., Barros L., Calhelha R.C., Ferreira I.C. (2019). Anthocyanin profile of elderberry juice: A natural-based bioactive colouring ingredient with potential food application. Molecules.

[9] Szalóki-Dorkó L., Stéger-Máté M., Abrankó L. (2015). Evaluation of colouring ability of main European elderberry (Sambucus nigra L.) varieties as potential resources of natural food colourants. Int. J. Food Sci. Technol..

[10] Domínguez R., Zhang L., Rocchetti G., Lucini L., Pateiro M., Munekata P.E., Lorenzo J.M. (2020). Elderberry (Sambucus nigra L.) as potential source of antioxidants. Characterization, optimization of extraction parameters and bioactive properties. Food Chem..

[11] Natić M., Pavlović A., Bosco F.L., Stanisavljević N., Zagorac D.D., Akšić M.F., Papetti A. (2019). Nutraceutical properties and phytochemical characterization of wild Serbian fruits. Eur. Food Res. Technol..

[12] Ürkek B., Şengül M., Akgül H.İ., Kotan T.E. (2019). Antioxidant activity, physiochemical and sensory characteristics of ice cream incorporated with sloe berry (Prunus spinosa L.). Int. J. Food Eng..

[13] Bal L.M., Meda V., Naik S.N., Satya S. (2011). Sea buckthorn berries: A potential source of valuable nutrients for nutraceuticals and cosmoceuticals. Food Res. Int..

[14] Routray W., Mishra H.N. (2011). Scientific and technical aspects of yogurt aroma and taste: A review. Compr. Rev. Food Sci. Food Saf..

[15] Baranowska M. (2006). Intensification of the synthesis of flavour compounds in yogurt by milk enrichment with their precursors. Pol. J. Food Nutr. Sci..

[16] Veberic R., Jakopic J., Stampar F., Schmitzer V. (2009). European elderberry (Sambucus nigra L.) rich in sugars, organic acids, anthocyanins and selected polyphenols. Food Chem..

[17] Cirlini M., Ricci A., Galaverna G., Lazzi C. (2020). Application of lactic acid fermentation to elderberry juice: Changes in acidic and glucidic fractions. LWT.

[18] Ricci A., Cirlini M., Levante A., Dall’Asta C., Galaverna G., Lazzi C. (2018). Volatile profile of elderberry juice: Effect of lactic acid fermentation using L. plantarum, L. rhamnosus and L. casei strains. Food Res. Int..

[19] Ranadheera C.S., Evans C.A., Adams M.C., Baines S.K. (2012). Probiotic viability and physico-chemical and sensory properties of plain and stirred fruit yogurts made from goat’s milk. Food Chem..

[20] Kailasapathy K., Harmstorf I., Phillips M. (2008). Survival of Lactobacillus acidophilus and Bifidobacterium animalis ssp. lactis in stirred fruit yogurts. LWT.

[21] Meybodi N.M., Mortazavian A.M., Arab M., Nematollahi A. (2020). Probiotic viability in yoghurt: A review of influencial factors. Int. Dairy J..

[22] Sun-Waterhouse D., Zhou J., Wadhwa S.S. (2013). Drinking yoghurts with berry polyphenols added before and after fermentation. Food Control.

[23] Gunenc A., Khoury C., Legault C., Mirrashed H., Rijke J., Hosseinian F. (2016). Seabuckthorn as a novel prebiotic source improves probiotic viability in yogurt. LWT.

[24] Terpou A., Gialleli A.I., Bosnea L., Kanellaki M., Koutinas A.A., Castro G.R. (2017). Novel cheese production by incorporation of sea buckthorn berries (Hippophae rhamnoides L.) supported probiotic cells. LWT.

[25] Terpou A., Papadaki A., Bosnea L., Kanellaki M., Kopsahelis N. (2019). Novel frozen yogurt production fortified with sea buckthorn berries and probiotics. LWT.

[26] Qureshi T.M., Nadeem M., Ahmad M.M., Hussain S., Rehman S., Shaukat A. (2017). Antioxidant potential of natural fruit flavored yogurt-a review. Pak. J. Agric. Res..

[27] Najgebauer-Lejko D., Sady M. (2015). Estimation of the antioxidant activity of the commercially available fermented milks. Acta Sci. Pol. Technol. Aliment..

[28] Zadernowski R., Naczk M., Czaplicki S., Rubinskiene M., Szałkiewicz M. (2005). Composition of phenolic acids in sea buckthorn (*Hippophae rhamnoides* L.) berries. J. AOCS.

[29] Cosmulescu S., Trandafir I., Nour V. (2017). Phenolic acids and flavonoids profiles of extracts from edible wild fuits and their antioxidant properties. Int. J. Food Prop..

[30] Trigueros L., Wojdyło A., Sendra E. (2014). Antioxidant activity and protein-polyphenol interactions in a pomegranate (*Punica granatum* L.) yoghurt. J. Agric. Food Chem..

[31] Karaaslan M., Ozden M., Vardin H., Turkoglu H. (2011). Phenolic fortification of yogurt using grape and callus extracts. LWT.

[32] Skrede G., Larsen V.B., Aaby K., Jørgensen A.S., Birkeland S.E. (2004). Antioxidative properties of commercial fruit preparations and stability of bilberry and black currant extracts in milk products. J. Food Sci..

[33] Gao X., Ohlander M., Jeppsson N., Björk L., Trajkovski V. (2000). Changes in antioxidant effects and their relationship to phytonutrients in fruits of sea buckthorn (*Hippophae rhamnoides* L.) during maturation. J. Agric. Food Chem..

[34] Gawai K.M., Mudgal S.P., Prajapati J.B., Shah N. (2017). Stabilizers, colorants, and exopolysaccharides in yogurt. Yogurt in Health and Disease Prevention.

[35] Du X., Myracle A.D. (2018). Development and evaluation of kefir products made with aronia or elderberry juice: Sensory and phytochemical characteristics. Int. Food Res. J..

[36] Wrolstad R.E., Durst R.W., Lee J. (2005). Tracking color and pigment changes in anthocyanin products. Trends Food Sci. Technol..

[37] Ścibisz I., Ziarno M., Mitek M. (2019). Color stability of fruit yogurt during storage. J. Food Sci. Technol..

[38] Christaki E. (2012). *Hippophae rhamnoides* L. (Sea Buckthorn): A potential source of nutraceuticals. Food Public Health.

[39] Sikora E., Bieniek M.I., Borczak B. (2013). Composition and antioxidant properties of fresh and frozen stored blackthorn fruits (*Prunus spinosa* L.). Acta Sci. Pol. Technol. Aliment..

[40] Sánchez L., Pérez M.D., Arana I. (2012). Physical properties of dairy products. Physical Properties of Foods: Novel Measurement Techniques and Applications.

[41] Amal A., Eman A., Nahla S.Z. (2016). Fruit flavored yogurt: Chemical, functional and rheological properties. Int. J. Environ. Agric. Res..

[42] Polish Standard: PN-75/A 86130 (1975). Mleko i Przetwory Mleczarskie—Napoje Mleczne—Metody Badań [Milk and Dairy Products—Fermented Milks—Analytical Methods].

[43] Association of Official Analytical Chemists (1985). Official Methods of Analysis.

[44] Polish Standard: PN-A-86061:2002/Az1:2006 (2006). Mleko i Przetwory Mleczne—Mleko Fermentowane [Milk and Dairy Products—Fermented Milks].

[45] Najgebauer-Lejko D., Sady M., Grega T., Walczycka M. (2011). The impact of tea supplementation on microflora, pH and antioxidant capacity of yoghurt. Int. Dairy J..

[46] Lee J., Durst R.W., Wrolstad R.E. (2005). Determination of total monomeric anthocyanin pigment content of fruit juices, beverages, natural colorants, and wines by the pH differential method: Collaborative study. J. AOAC Int..

[47] Klimczak I., Małecka M., Szlachta M., Gliszczyńska-Świgło A. (2007). Effect of storage on the content of polyphenols, vitamin C and the antioxidant activity of orange juices. J. Food Compos. Anal..

[48] ISO 7889/IDF 117 (2003). Yogurt—Enumeration of Characteristic Microorganisms-Colony-Count Technique at 37 Degrees C.

[49] IDF Standard 149A (1997). Dairy Starter Cultures of Lactic Acid Bacteria (LAB). Standardof Identity.

[50] Dave R.I., Shah N.P. (1996). Evaluation of media for selective enumeration of *Streptococcus thermophilus*, *Lactobacillus delbrueckii* ssp. *bulgaricus*, *Lactobacillus acidophilus*, and bifidobacteria. J. Dairy Sci..

[51] IDF 94:2004 (2004). Milk and Milk Products—Enumeration of Colony-Forming Units of Yeasts And/Or Moulds—Colony-Count Technique at 25 Degrees C.

[52] Lees G.J., Jago G.R. (1969). Methods for the estimation of acetaldehyde in cultured dairy products. Aust. J. Dairy Technol..

[53] Pien J. (1974). Etude de beurre. Tech. Lait..

